# Stability of different fixation methods after reduction malarplasty under average and maximum masticatory forces: a finite element analysis

**DOI:** 10.1186/s12938-023-01098-8

**Published:** 2023-04-21

**Authors:** Mohammed Qasem Al-Watary, Heyou Gao, Libin Song, Yingyou He, Yiyuan Wei, Jihua Li

**Affiliations:** State Key Laboratory of Oral Diseases and National Clinical Research Center for Oral Diseases, Center of Orthognathic and TMJ Surgery, West China Hospital of Stomatology, Sichuan University, Chengdu, People’s Republic of China

**Keywords:** Reduction malarplasty, Stability, Zygomatic body fixation methods, Finite element methods, Zygomatic arch fixation methods

## Abstract

**Background:**

Although titanium plates/screws are effective fixation methods (FM) after L-shaped osteotomy reduction malarplasty (LORM), the ideal FM remains controversial. This first finite element analysis (FEA) aimed to study the effect of various zygomatic body/zygomatic arch FM combinations and their placement vectors on the zygoma complex stability after virtual LORM under the effect of both average (150 N/mm^2^) and maximum (750 N/mm^2^) forces and three-dimensional (3D) mapping of stress and strain parameters distribution over the zygomatic bone, fixation methods, and total model.

**Results:**

The fixation methods about the short-arm of the L-shaped osteotomy showed lower stress, strain, and displacement values than those across the long-arm osteotomy site. Combined with any zygomatic arch fixation methods (ZAFm), the two bicortical screws group (2LS) on the zygomatic body osteotomy site resulted in smaller displacements and the lowest zygoma bone stress and displacement when combined with Mortice–Tenon structure (MT) as zygomatic arch fixation method. Applied forces caused statistically significant differences in zygomatic bone stress (*P* < 0.001 and *P* = 0.001) and displacement (*P* = 0.001 and *P* = 0.002).

**Conclusion:**

All FMs both on the zygomatic body and zygomatic arch provide adequate zygomatic complex stability after LORM. The 2LS group showed better resistance than rectangular plate (RP) and square plate (SP) with lower stress concentrations. The L-shaped plate with short-wing on the maxilla (LPwM) is more stable than having the short-wing on the zygoma bone (LPwZ). Future prospective clinical studies are required to validate the current findings.

## Background

The zygomatic bone is a prominent midface structure that is crucial for three-dimensional facial aesthetic appearance. It shows racial characteristics by determining the lateral face's height, width, and projection [1]. Orientals are characterised by high and overly protruding zygomas that upset the facial units’ harmony with rough, aggressive, and masculine appearance [1–5]. Therefore, reduction malarplasty (RM) is commonly practised in East Asia [6]. Over the past three decades, several surgical techniques have been introduced, with progressive advances in simplicity, safety, and aesthetic outcomes [2, 3, 7–14]. Most of these studies were focused on the osteotomy design on both the zygomatic body (ZB) and the zygomatic arch (ZA). Currently, L-shaped osteotomy RM (LORM) consisting of intraoral L-shaped osteotomy (LO) with simultaneous short-sideburn incision is most popular because of its advantages: short surgical time, small scar, low risk of facial nerve injury, and fast recovery [6, 12, 14–18]. The optimal RM procedure should fulfil the following goals: midfacial width reduction, flattening of the zygomatic prominence, preservation of the natural malar curvature, nonvisible scar, and zygomatic complex sustained height as proposed by Nakanishi et al. [10]. Of equal importance is maintaining intimate bone-to-bone contact on the zygomatic body’s osteotomy line and zygomatic complex (body and arch) fixation without bony dehiscence [19]. The fundamental problem of LORM is the instability due to poor fixation, which is exaggerated by powerful masseteric inferior pulling action [19, 20]. Not surprisingly, the literature revealed that postoperative zygomatic complex mobility is conductive to complications such as malunion or nonunion with resultant unfavourable outcomes such as sagging of the cheek, facial asymmetry, malar depression, and restricted jaw movement [16, 21–27]. All these outcomes mandate corrective surgical procedures through aggressive approaches such as the bicoronal flap approach that could result in major morbidities like big facial scar, hair loss, and facial nerve injury [16, 22, 24]. Therefore, prevention of these unfoavourable outcomes should be kept in surgeons mind. This prevention could be achieved by choosing robust internal fixation methods after establishing an intimate bone-to-bone contact in the L-shaped osteotomy region. Of the same importance, is the fixation methods’ placement vector about the L-shaped osteotomy line. To the best of our knowledge, there are no comparative studies showed the effect of combined zygomatic body fixation methods (ZBFm) with zygomatic arch fixation methods (ZAFm) on the total stability of the zygomatic complex after L-shaped osteotomy reduction malarplasty. Most previous studies focused on either the fixation methods on the zygomatic body or on the zygomatic arch separately. Kim et al. [15] demonstrated in their experiment on skull replicate samples that it is better to locate the fixation point at a higher level in the medial part of the zygomatic body, closer to the lateral orbital rim. According to studies by Baek et al. [16, 23, 24] and Lee and Lee [22], the intraoral approach does not allow fixation of the lateral orbital rim side, it cannot withstand the strong action of the masseter muscle, which may cause the unfavourable outcomes mentioned above. On the other hand, Hwang et al. [19] and Wang et al. [21] explained the effect of zygomatic arch fixation method on the stability of RM outcomes despite the fixation methods used on ZB region.

Hence, there is no consensus regarding the optimum FM combinations and best placement vector that will provide long-term stable outcomes. Therefore, choosing ZB/ZA fixation method combinations and their placement vector is still controversial among surgeons. One reason such controversies persist is that the basic biomechanics of the zygomatic complex region are not well understood [28–30].

FEA is a numerical approach simulating the dynamics of physical objects with confirmed benefits in evaluating facial fracture plating techniques [31, 32]. It has the following advantages: graphic visualisation of unseen regions, precise model simulation of perioperative behaviour, time savings, cost-effectiveness, repeatability, and various clinical scenario simulations through variations in force application point, magnitude, and direction [31].

However, the fixation method efficiency after LORM has never been studied using 3D-FEA. Therefore, this first time FEA study aimed to investigate the stability of commonly used zygomatic body and zygomatic arch fixation method combinations under normal (150 N/mm^2^) and maximum (750 N/mm^2^) masticatory forces and 3D-mapping of the stress and strain distribution over zygoma bone, FM, and the overall model after virtual LORM. In addition, we studied the effect of placement vector of L-shaped plate through comparing the stability and the stress distribution pattern when the short wing fixated on the zygoma (LPwZ) versus fixated on the maxilla (LPwM). Therefore, the results of this study can serve as an evidence-based guide for the selection of stable ZB/ZA fixation method combinations and the optimal placement vectors for these combinations. With such evidence, disputes among surgeons in this area will be resolved for good clinical practice, which will positively impact the health of patients as well as the profession and reputation of surgeons. Furthermore, the present results can be applied to industrial fixation methods (titanium plates and screws). Therefore, stronger titanium plates can be produced by strengthening the weak regions that appeared in the stress–strain 3D-mapping under loading conditions.

## Results

### Stress analysis

**Fig. 1:**
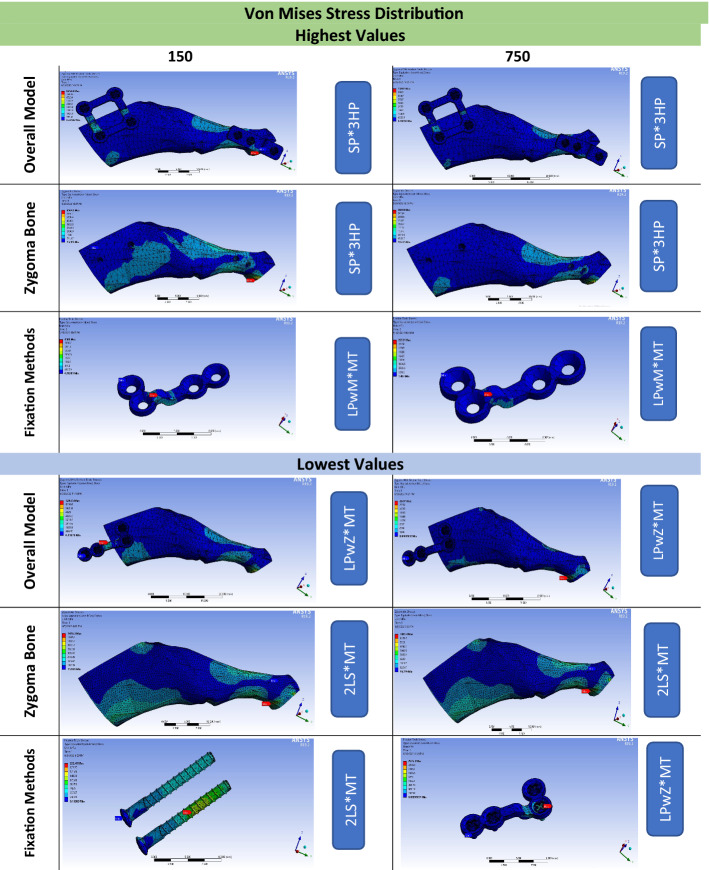
Colour-coded spectrum demonstrating the highest and lowest Von Mises stress distribution over the overall model, zygoma bone, and fixation methods under average (150 N/mm^2^) and maximum (750 N/mm^2^) masticatory forces. *SP* square plate, *3HP* 3-hole-plate, *LPwM* L-shaped plate with a short wing on the maxilla, *MT* Mortice–Tenon, *LPwZ* L-shaped plate with a short wing on the zygoma, *2LS* 2 long bicortical screws

Under 150 and 750 N/mm^2^, the highest stress values of the overall model (373.58 and 1817.5, respectively) and zygoma bone (604.1 and 2934.6, respectively) belonged to the SP*3HP combination, as shown in the colour-coded diagram (Fig. 1). The LPwZ*MT combination recorded the lowest values of the overall model under both forces (58.714 and 157.39, respectively), while the 2LS*MT combination showed zygoma bone’s lowest values (149.73 and 748.66, respectively). Regarding the stress distribution over the fixation methods, the highest values were demonstrated by the LPwM*MT combination under both forces (265.53 and 1325.2, respectively). However, the lowest value under 150 N/mm^2^ was shown by the 2LS*MT combination (42.566), which was increased almost four times under 750 N/mm^2^ with the LPwZ*MT combination (163.03) (Table 1).

**Table 1:** The average and maximum values of stress concentration associated with tested fixation methods under normal (150 N/mm^2^) and maximum (750 N/mm^2^) masticatory forces for zygoma bone, fixation methods, and overall model

ZBFm	ZAFm	Load	Stress					
			Zygoma bone		Fixation methods		Overall model	
			Aver	Max	Aver	Max	Aver	Max
LPwZ	3HP	150N	499.760	6272.200	158.200	11,705.000	278.000	11,705.000
		**750N**	**2431.300**	**35,518.000**	**863.400**	**59,659.000**	**1413.300**	**59,659.000**
	MT	150N	473.33	5963.5	145.28	7243.5	58.714	7243.5
		**750N**	**1520.7**	**28,791**	**163.03**	**2975.3**	**157.39**	**28,791**
	SS	150N	494.290	5008.300	129.200	9886.800	291.960	9886.800
		**750N**	**2449.000**	**85,299.000**	**660.610**	**40,412.000**	**1457.900**	**85,299.000**
LPwM	3HP	150N	271.920	2386.700	191.640	4342.300	185.650	4342.300
		**750N**	**1320.300**	**13,655.000**	**966.650**	**29,116.000**	**924.030**	**29,116.000**
	MT	150N	192.620	2484.800	265.530	4377.200	146.000	4377.200
		**750N**	**952.090**	**14,809.000**	**1325.200**	**29,441.000**	**738.600**	**29,441.000**
	SS	150N	190.460	2016.500	45.924	630.230	87.192	2261.700
		**750N**	**952.280**	**10,083.000**	**229.620**	**3151.100**	**435.960**	**11,309.000**
LPLS	3HP	150N	215.680	2411.800	62.861	3249.300	103.280	3249.300
		**750N**	**1078.400**	**12,059.000**	**314.300**	**16,247.000**	**516.380**	**16,247.000**
	MT	150N	452.800	6913.900	120.410	7964.400	266.480	7964.400
		**750N**	**2228.300**	**42,793.000**	**508.880**	**38,255.000**	**699.500**	**7081.400**
	SS	150N	219.230	2095.800	43.506	2818.000	102.420	2818.000
		**750N**	**1096.100**	**10,479.000**	**217.530**	**14,090.000**	**512.120**	**14,090.000**
2LS	3HP	150N	183.210	2164.000	73.198	855.760	127.960	2164.000
		**750N**	**916.030**	**10,820.000**	**365.990**	**4278.800**	**639.800**	**10,820.000**
	MT	150N	149.730	1416.300	42.566	222.470	139.900	1416.300
		**750N**	**748.660**	**7081.400**	**212.830**	**1112.300**	**699.500**	**7081.400**
	SS	150N	170.700	11,297.000	108.280	3379.100	162.900	11,297.000
		**750N**	**853.520**	**56,486.000**	**541.420**	**16,896.000**	**814.490**	**56,486.000**
RP	3HP	150N	514.460	6526.900	190.540	10,344.000	289.300	10,344.000
		**750N**	**2488.000**	**41,055.000**	**941.420**	**53,717.000**	**1413.000**	**53,717.000**
	MT	150N	585.770	6965.000	228.720	8423.500	365.970	8423.500
		**750N**	**2901.700**	**34,086.000**	**1121.000**	**28,839.000**	**1805.500**	**34,086.000**
	SS	150N	578.760	17,843.000	171.320	7826.100	324.130	17,843.000
		**750N**	**2873.600**	**168,490.000**	**901.810**	**46,250.000**	**1641.300**	**168,490.000**
SP	3HP	150N	604.100	6564.100	249.600	8650.800	373.580	8650.800
		**750N**	**2934.600**	**38,410.000**	**1216.800**	**55,802.000**	**1817.500**	**55,802.000**
	MT	150N	508.290	6632.100	181.550	9107.200	335.010	9107.200
		**750N**	**2587.200**	**37,269.000**	**936.650**	**48,460.000**	**1711.900**	**48,460.000**
	SS	150N	474.050	11,878.000	136.580	8936.500	287.750	11,878.000
		**750N**	**1277.900**	**17,383.000**	**190.020**	**3103.300**	**1030.300**	**17,383.000**

The bold values represent the values under the maximum masticatory forces (750 N/mm^2^)

*ZBFm* zygomatic body fixation methods, *ZAFm* zygomatic arch fixation methods, *Aver* average, *Max* maximum, *LPwZ* L-shaped plate with a short wing on the zygoma, *LPwM* L-shaped plate with a short wing on the maxilla, *LPLS* L-shaped plate with one long bicortical screw, *2LS* two long bicortical screws, *RP* rectangular plate, *SP* square plate, *3HP* 3-hole plate, *MT* Mortice–Tenon, *SS* short screw

**Fig. 2:**
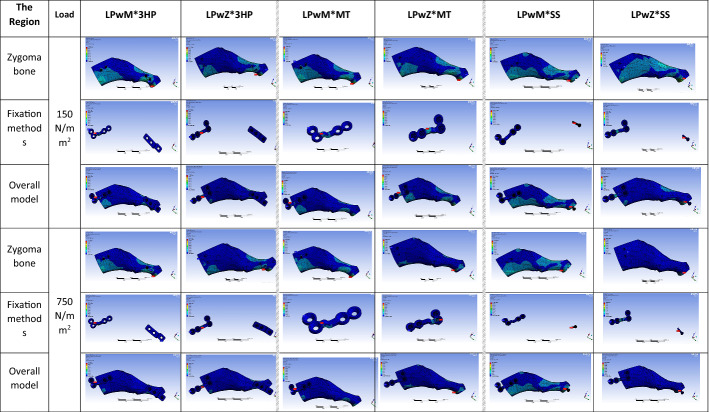
Colour-coded spectrum demonstrating the zygoma bone, fixation methods, and overall model stress concentration associated with LPwM compared to LPwZ as zygomatic body fixation methods combined with 3-hole plate, Mortice–Tenon, and short screw as zygomatic arch fixation methods under normal (150 N/mm^2^) and maximum (750 N/mm^2^) masticatory forces

The colour-coded diagram (Fig. 2) showed that LPwM outperformed LPwZ under both forces. The ANOVA test revealed that all ZBFm groups under both forces showed statistically significant differences in stress distribution over zygoma bone (*P* < 0.001 and P = 0.001, respectively) and the overall model (*P* < 0.001 and *P* = 0.001, respectively) with nonsignificant differences over fixation methods (P = 0.071 and P = 0.173, respectively). ZAFm had nonsignificant differences in all measuring parameters.

### Strain analysis

The SP*3HP combination recorded the highest strain values for the overall model and zygoma bone under 150 N/mm^2^ (0.00283 and 0.00311, respectively) and 750 N/mm^2^ (0.01382 and 0.01512, respectively). The lowest values for the overall model were shown by the LPLS*SS combination (150 N/mm^2^ = 0.00068 and 750 N/mm^2^ = 0.00340), while zygoma bone values were revealed by 2LS*MT (150 N/mm^2^ = 0.00076 and 750 N/mm^2^ = 0.00378). The highest values for fixation methods under both forces were shown by the LPwM*MT combination (150 N/mm^2^ = 0.00287 and 750 N/mm^2^ = 0.01453, respectively), while the lowest values were recorded by 2LS*MT (150 N/mm^2^ = 0.00044) and LPwZ*MT (750 N/mm^2^ = 0.00170). The ANOVA test among ZBFm at 150 N/mm^2^ revealed a statistically significant difference for zygoma bone (*P* = 0.000) and fixation methods (*P* = 0.039), while the overall model had a statistically nonsignificant difference (*P* = 0.394). In contrast, under 750 N/mm^2^, the differences were statistically significant for both the overall model (*P* = 0.032) and zygoma bone (*P* = 0.013), but for the fixation methods, they were statistically nonsignificant (*P* = 0.241). Regarding the ZAF methods, both forces yielded statistically nonsignificant differences for all parameters (Table 2).

**Table 2:** The average and maximum values of strain distribution associated with tested fixation methods under normal (150 N/mm^2^) and maximum (750 N/mm^2^) masticatory forces for zygoma bone, fixation methods, and overall model

ZBFm	ZAFm	Load	STRAIN					
			Zygoma bone		Fixation methods		Overall model	
			Aver	Max	Aver	Max	Aver	Max
LPwZ	3HP	150N	0.00257	0.03494	0.00170	0.12810	0.00201	0.12810
		**750N**	**0.01252**	**0.20340**	**0.00930**	**0.64680**	**0.01043**	**0.64680**
	MT	150N	0.00243	0.03484	0.00154	0.07443	0.00196	0.07443
		**750N**	**0.00788**	**0.14450**	**0.00170**	**0.03662**	**0.00463**	**0.14450**
	SS	150N	0.00254	0.02845	0.00138	0.09737	0.00190	0.09737
		**750N**	**0.01260**	**0.44650**	**0.00705**	**0.39970**	**0.00952**	**0.44650**
LPwM	3HP	150N	0.00138	0.01237	0.00211	0.04462	0.00119	0.04462
		**750N**	**0.00671**	**0.07091**	**0.01074**	**0.30658**	**0.00603**	**0.30658**
	MT	150N	0.00099	0.01381	0.00287	0.04499	0.00097	0.04499
		**750N**	**0.00487**	**0.08011**	**0.01453**	**0.31009**	**0.00498**	**0.31009**
	SS	150N	0.00099	0.01054	0.00077	0.02416	0.00079	0.02416
		**750N**	**0.00494**	**0.05272**	**0.00255**	**0.03251**	**0.00396**	**0.12078**
LPLS	3HP	150N	0.00111	0.01226	0.00065	0.04273	0.00077	0.04273
		**750N**	**0.00554**	**0.06131**	**0.00325**	**0.21360**	**0.00385**	**0.21360**
	MT	150N	0.00233	0.03840	0.00130	0.09670	0.00176	0.09670
		**750N**	**0.01149**	**0.24750**	**0.00557**	**0.47730**	**0.00817**	**0.47732**
	SS	150N	0.00113	0.01090	0.00046	0.02804	0.00068	0.02804
		**750N**	**0.00562**	**0.05448**	**0.00228**	**0.14020**	**0.00340**	**0.14020**
2LS	3HP	150N	0.00094	0.01097	0.00078	0.00940	0.00086	0.01097
		**750N**	**0.00472**	**0.05484**	**0.00391**	**0.04701**	**0.00431**	**0.05484**
	MT	150N	0.00076	0.00748	0.00044	0.00218	0.00073	0.00748
		**750N**	**0.00378**	**0.03739**	**0.00220**	**0.01092**	**0.00364**	**0.03739**
	SS	150N	0.00170	0.11417	0.00118	0.03794	0.00163	0.11417
		**750N**	**0.00849**	**0.57087**	**0.00591**	**0.18972**	**0.00817**	**0.57087**
RP	3HP	150N	0.00265	0.03870	0.00203	0.11527	0.00222	0.11527
		**750N**	**0.01282**	**0.24564**	**0.01007**	**0.59082**	**0.01091**	**0.59082**
	MT	150N	0.00301	0.03505	0.00248	0.09089	0.00268	0.09089
		**750N**	**0.01495**	**0.19711**	**0.01213**	**0.30558**	**0.01322**	**0.30558**
	SS	150N	0.00298	0.08922	0.00183	0.08277	0.00226	0.08922
		**750N**	**0.01488**	**0.90362**	**0.00965**	**0.48291**	**0.01161**	**0.90362**
SP	3HP	150N	0.00311	0.03771	0.00268	0.09765	0.00283	0.09765
		**750N**	**0.01512**	**0.21167**	**0.01313**	**0.61798**	**0.01382**	**0.61798**
	MT	150N	0.00262	0.03780	0.00199	0.10692	0.00228	0.10692
		**750N**	**0.01335**	**0.23322**	**0.01026**	**0.56629**	**0.01171**	**0.56629**
	SS	150N	0.00244	0.05971	0.00151	0.11082	0.00193	0.11082
		**750N**	**0.00646**	**0.09474**	**0.00198**	**0.03319**	**0.00544**	**0.09474**

Bold values represent the values under the maximum masticatory forces (750 N/mm^2^)

### Displacement analysis

The highest displacement values for the overall model under both forces were associated with the 2LS*SS combination (150 N/mm^2^ = 0.049 mm and 750 N/mm^2^ = 0.245 mm) (Table 3).

**Table 3:** The average and maximum values of displacement values associated with tested fixation methods under normal (150 N/mm^2^) and maximum (750 N/mm^2^) masticatory forces for zygoma bone, fixation methods, and overall model

ZBFm	ZAFm	Load	Displacement					
			Zygoma bone		Fixation methods		Overall model	
			Aver	Max	Aver	Max	Aver	Max
LPwZ	3HP	150N	0.183	0.421	0.085	0.298	0.034	0.421
		750N	**0.832**	**1.947**	**0.383**	**1.385**	**0.152**	**1.947**
	MT	150N	0.183	0.421	0.085	0.298	0.025	0.430
		750N	**0.832**	**1.947**	**0.383**	**1.385**	**0.020**	**0.490**
	SS	150N	0.167	0.266	0.060	0.184	0.024	0.266
		750N	**0.787**	**1.250**	**0.291**	**0.901**	**0.115**	**1.250**
LPwM	3HP	150N	0.108	0.226	0.064	0.189	0.040	0.226
		750N	**0.494**	**1.029**	**0.293**	**0.890**	**0.184**	**1.029**
	MT	150N	0.105	0.227	0.038	0.083	0.028	0.227
		750N	**0.477**	**1.041**	**0.222**	**0.511**	**0.127**	**1.041**
	SS	150N	0.060	0.122	0.060	0.122	0.018	0.122
		750N	**0.298**	**0.610**	**0.298**	**0.610**	**0.090**	**0.610**
LPLS	3HP	150N	0.066	0.132	0.039	0.145	0.020	0.145
		750N	**0.332**	**0.659**	**0.193**	**0.727**	**0.102**	**0.727**
	MT	150N	0.182	0.435	0.050	0.136	0.027	0.435
		750N	**0.812**	**2.036**	**0.195**	**0.569**	**0.115**	**2.036**
	SS	150N	0.059	0.132	0.019	0.040	0.011	0.132
		750N	**0.294**	**0.662**	**0.095**	**0.200**	**0.057**	**0.662**
2LS	3HP	150N	0.047	0.105	0.041	0.115	0.015	0.115
		750N	**0.237**	**0.523**	**0.205**	**0.575**	**0.074**	**0.575**
	MT	150N	0.037	0.087	0.011	0.019	0.020	0.087
		750N	**0.187**	**0.435**	**0.057**	**0.095**	**0.101**	**0.435**
	SS	150N	0.100	0.272	0.023	0.164	0.049	0.272
		750N	**0.502**	**1.360**	**0.116**	**0.818**	**0.245**	**1.360**
RP	3HP	150N	0.198	0.443	0.087	0.303	0.039	0.443
		750N	**0.897**	**2.086**	**0.383**	**1.402**	**0.173**	**2.086**
	MT	150N	0.244	0.452	0.112	0.244	0.039	0.452
		750N	**1.135**	**2.109**	**0.504**	**1.141**	**0.178**	**2.109**
	SS	150N	0.193	0.320	0.075	0.208	0.032	0.320
		750N	**0.903**	**1.543**	**0.351**	**1.008**	**0.152**	**1.543**
SP	3HP	150N	0.239	0.453	0.113	0.317	0.044	0.453
		750N	**1.092**	**2.069**	**0.502**	**1.471**	**0.197**	**2.069**
	MT	150N	0.234	0.523	0.104	0.242	0.034	0.523
		750N	**1.099**	**2.366**	**0.482**	**1.183**	**0.161**	**2.366**
	SS	150N	0.159	0.257	0.054	0.174	0.023	0.257
		750N	**0.358**	**0.843**	**0.097**	**0.237**	**0.136**	**0.843**

Bold values represent the values under the maximum masticatory forces (750 N/mm^2^)

**Fig. 3:**
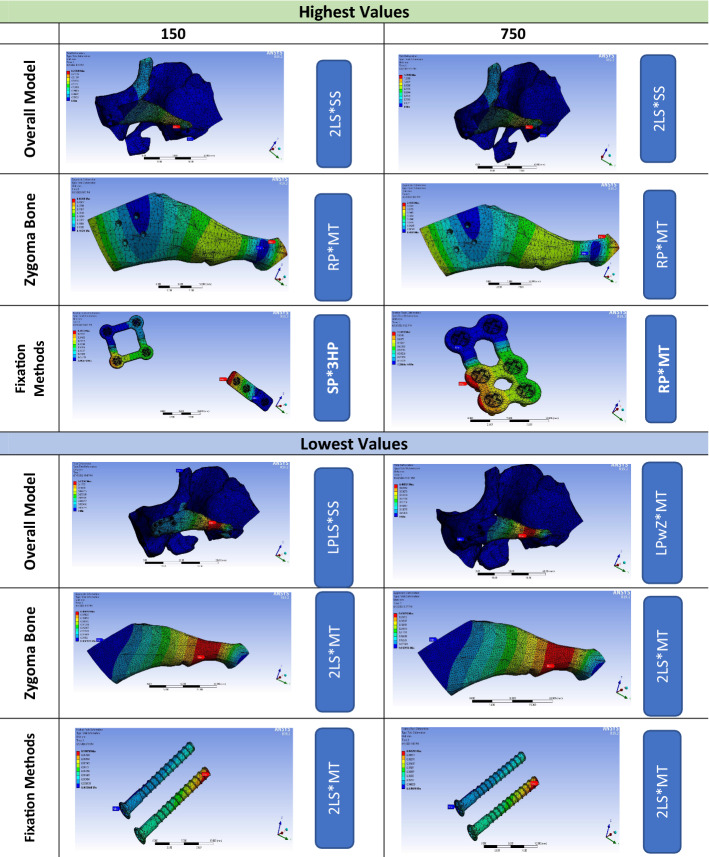
Colour-coded spectrum demonstrating the highest and lowest displacement values of the overall model, zygoma bone, and fixation methods under average (150 N/mm^2^) and maximum (750 N/mm^2^) masticatory forces

**Fig. 4:**
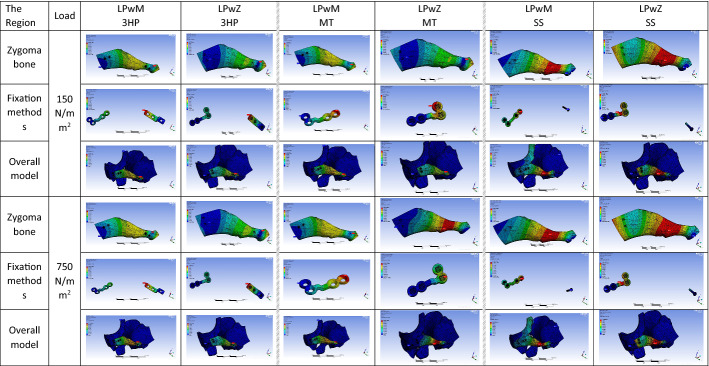
Colour-coded spectrum demonstrating the zygoma bone, fixation methods, and overall model displacement values associated with LPwM compared to LPwZ as zygomatic body fixation methods combined with 3-hole plate, Mortice–Tenon, and short screw as zygomatic arch fixation methods under normal (150 N/mm^2^) and maximum (750 N/mm^2^) masticatory forces

However, LPLS*SS recorded the lowest value (0.011 mm) under 150 N/mm^2^, while under 750 N/mm^2^, almost double the value (0.020 mm) was associated with the LPwZ*MT combination. The magnitude of the displacement related to the RP*MT combination, as illustrated by a colour-coded spectrum, was the highest value under both forces for the zygoma bone (150 N/mm^2^ = 0.244 mm and 750 N/mm^2^ = 1.135 mm) and the fixation methods’ displacement under 750 N/mm^2^ (0.504 mm) (Fig. 3). In addition, the highest fixation method displacement value under 150 N/mm^2^ was recorded by the SP*3HP combination (0.113 mm). On the other hand, the lowest displacement values under both forces for zygoma bone were 0.037 mm under 150 N/mm^2^ and 0.187 mm under 750 N/mm^2^, and the lowest values for fixation methods (150 N/mm^2^ = 0.011 mm and 750 N/mm^2^ = 0.057 mm) were associated with the 2LS*MT combination. In addition, the colour-coded diagram (Fig. 4) showed the superiority of LPwM over LPwZ in holding the zygoma complex against the masseteric simulation forces. The zygomatic bone and fixation methods showed statistically significant differences under 150 N/mm^2^ (*P* = 0.001 and *P* = 0.004, respectively) and 750 N/mm^2^ (*P* = 0.020 and *P* = 0.040, respectively). At the same time, the overall model failed to show statistically significant differences (*P* = 0.491 and *P* = 0.403), as revealed by ANOVA. At the same time, all ZAFM groups’ displacements were statistically nonsignificant differences.

## Discussion

The FEA is a powerful tool that has been used extensively in structural analysis of facial skeletons that allows detailed visualisation of where structures bend or twist and indicates the distribution of stresses and displacements [31, 32]. As a result of the computing advancement, we are increasingly turning to virtual analysis and tools tailored to our needs to test the mechanical properties of facial soft and hard tissues and osteosynthesis materials, repetitively, safely, and cost-effectively, which are all met by FEA [33, 34]. Herein, we innovatively implemented the 3D-FEA to map the stress distribution over the most commonly used FM after LORM, which enabled the prediction of areas more susceptible to fracture under simulated masticatory forces.

Generally, the current findings showed that all ZBFm*ZAFm combinations were adequately resistant to zygoma bone displacement, as all records were below 2.5 mm, which from our clinical experience, is not recognised clinically and did not contribute to any complications that warrant reoperation [21].

The highest stress associated with RP and SP combined with any ZAFm over the overall model and zygoma bone could be explained by two points. First, their location was near the site of the maximum masseteric effect along the L-shaped osteotomy line’s long-arm. Second, the thick bone in this region tolerated longer screws, providing more bone surface contact and higher resistance to the masseter muscle. Interestingly, under masseteric action simulated forces, although, among the ZBFm groups, the maximum zygoma bone displacement did not reach above 2.4 mm, RP and SP showed the highest values. This may be due to the parallelism of the plate placement vector with the masseter muscle's vector, which may enhance the muscle's inferior displacement simulated forces. In addition, their highest stress concentration, as shown by the colour-coded diagram, could lead to either screw loosening or plate bridge deformities, as revealed by the high strain values. Such findings could not be explored without FEA help, as they cannot be understood or entirely revealed by in vitro biomechanical studies [35, 36]. Although it seems that RP and SP recorded parameters (stress, strain, and displacement) were higher than other ZBFm groups, all of them still showed good resistance against the inferior displacement. In line with Kim et al. [15] biomechanical study, placing the fixation tools at a higher level near the orbital rim provides more favourable outcomes. Baek et al. [16] also align with these findings. They proposed that placement of the fixation methods at a higher level across the osteotomy line would provide more support against the displacing forces.

Another interesting finding is the effect of changing the placement vector on the FMs’ performance. This is obvious in the amount of resistance the single LP provided in different placement vectors. Having the short-wing fixed to the maxilla (fixed bone), the stress and displacement values of the zygoma bone were almost 50% lower than when fixed to the zygoma (mobile bone). This could have two explanations: the anti-rotation provided by the short-wing vertically arranged screws over the maxilla bone offers less stress and better resistance. Second, the plate's perpendicular placement vector across the short-arm allows support of the greater force. In addition, the distance between the short-wing and the osteotomy line is short, resulting in a higher fixation level with a smaller external rotating force [15].

Our results could explain the outperformance of the 2LS and LPLS groups, holding the zygoma complex (body and arch) more stable due to their double bridges of fixation. These findings concord with Baek et al. [16] who emphasised that at least a two-bridge fixation method on the ZB should be placed to provide adequate support. Hence, the 2LS group is highly recommended when there is a good amount of bone, as it is strong enough to hold the freed zygoma against the rotational masseter muscle force. Furthermore, from our clinical experience, they require less surgical placement time; postoperatively, they are less palpable, no migration could occur compared to plates, and they are less costly.

On the other hand, the ZAF methods after RM also have no standard protocol. Nevertheless, from the literature, the most commonly used methods are either MT structure or titanium plates and screws to ensure good bony consolidation [7, 14, 19, 37]. The authors first proposed the MT structure in 2014, in which the Mortice is formed by the ZA free end and the Tenon by the gap between the zygomatic process laterally and temporal bone medially [7, 21].

The current study confirmed the importance of ZBFm and ZAFm combined stability on freed zygomatic complex (body and arch) stability after RM. Meanwhile, they showed the effect of each type of ZAFm on the stress, strain, and displacement values over the zygomatic complex (body and arch). Furthermore, the findings showed that all ZAF methods performed well against the inferior displacement exerted by the masseter muscle simulated by the two forces (150 N/mm^2^ and 750 N/mm^2^).

From all these findings, we appreciate that FEA can vividly show the dynamic behaviour of the zygomatic complex under masseter muscle action and reveal the outcomes of this biomechanical interaction on the implanted plates and screws. Additionally, it is helpful to analyse the stress concentration on various parts of the zygomatic complex incorporating the fixation methods across the osteotomy lines. Therefore, these findings could guide surgeons in choosing the best type of fixation method and the optimum placement vector that provides better stability. In addition, industrial-wise, more robust plates could be produced by strengthening the weak areas that appeared on the stress–strain mapping under loading conditions. This is one of the strong points of the current study. Furthermore, we have studied, for the first time, the ability of 18 ZBFm* ZAFm combinations to withstand the inferiorly directed masseteric force, which was simulated by the loaded forces (the average (150 N/mm^2^) and voluntary maximum (750 N/mm^2^) masticatory forces after RM. However, FEA is just a method that simulates real physical conditions; thus, it cannot be completely accurate [32, 38]. Therefore, the findings of this FEA study need to be validated by conducting a prospective clinical study comparing the long-term effect of the same fixation methods studied here on the stability of the zygomatic complex (body and arch) after LORM.

## Conclusion

The findings of this first FE simulation suggest that all ZBFm*ZAFm combinations after LORM can provide adequate stability. RP and SP combined with any form of ZAFm did not differ significantly from 2LS, LPLS, or LPwM concerning zygoma bone displacement. The 2LS group showed better resistance with less stress concentrations. The single L-shaped plate will be more stable if its short-wing is fixed on the maxilla. Future studies validating the current findings are recommended.

## Materials and methods

### FE model creation and virtual surgery

Preoperative CT of a 26-year-old healthy consented female who underwent LORM to correct prominent zygomas was used to produce the virtual 3D zygoma complex model. The research committees at West China Hospital of Stomatology and Sichuan University approved this study (WCHS-CRSE-2022-103).

The Digital Imaging and Communications in Medicine (DICOM) format of the CT with 0.5 mm thickness was imported to 3D-Slicer software to extract the CT images and select the midface bone using the Hounsfield threshold (785.99 to 3071.00) to create a standard tessellation language file (STL) format (https://www.slicer.org) [39]. The 3D model was constructed in all three spatial planes of axial, coronal, and sagittal by manual segregation for better accuracy with a slice thickness of 0.5 mm. The STL file was edited in PTC Creo 4.0 M010 2016 (PTC, Boston, Massachusetts) software (https://www.ptc.com) to create FEA solid model, maintaining only the left zygoma complex bone with part of lateral and inferior orbital rims and posterolateral maxilla anteriorly, articular tubercle posteriorly, and temporal bone medially to fasten the calculation process.

**Fig. 5:**
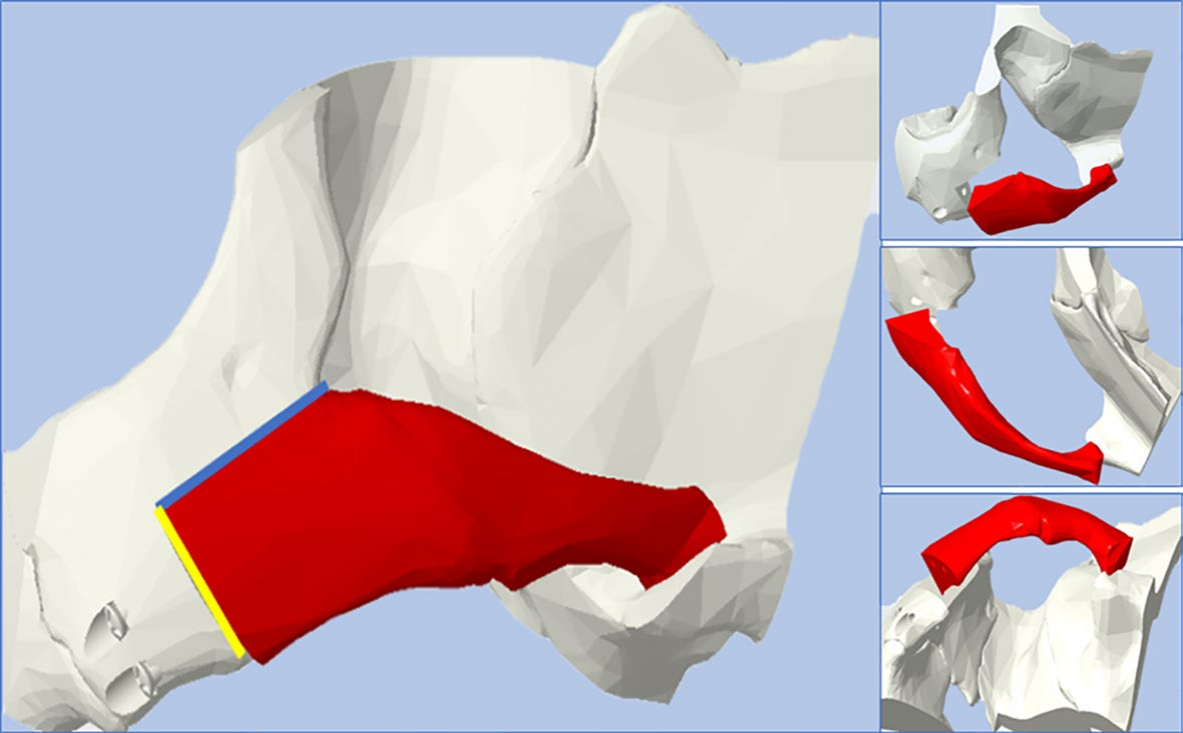
L-shaped osteotomy reduction malarplasty was carried out virtually resulting in two separated segments: zygomatic complex (body and arch: red labelled) and boundaries bones (white labelled). Blue line: long arm, yellow line: short arm. **A**: 45° view, **B** superior view, **C** inferior view

In PTC Creo 4.0, a virtual LORM procedure based on outlines drawn by a senior surgeon [21] was conducted, resulting in a freed zygomatic complex and stable surrounding borders (Fig. 5). The whole complex was then repositioned superomedially and posteriorly, guided by anterior bone-to-bone contact using the software's segmentation function.

**Fig. 6:**
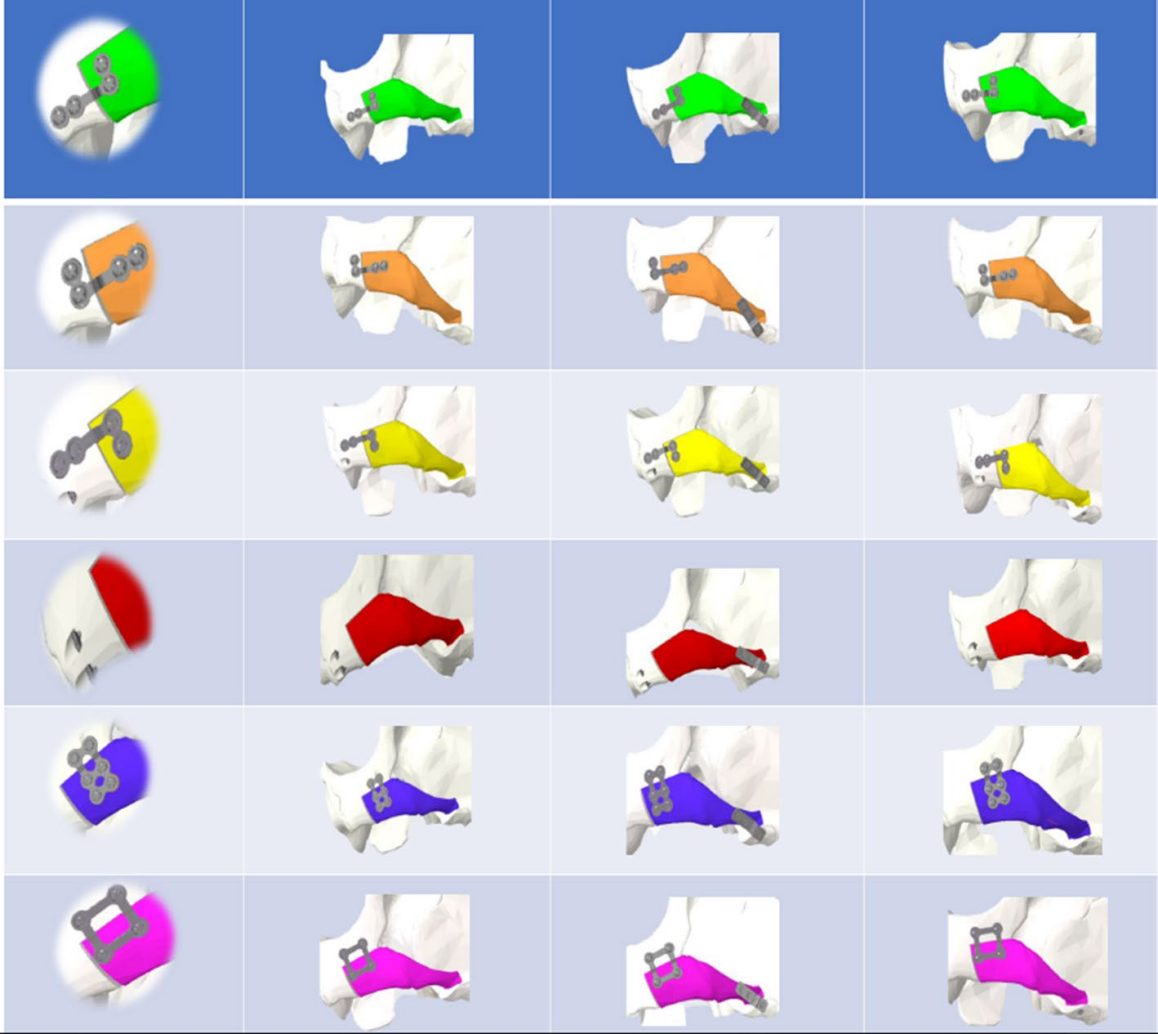
Models with fixation techniques across the short arm after virtual reduction malarplasty (**A**–**D**) and across the long arm (**E**, **F**): zygomatic body fixation methods (frontal view): **A** L-shaped plate with the short wing on the zygoma, **B** L-shaped plate with the short wing on the maxilla, **C** L-shaped plate with one bicortical screw **D** Two bicortical screws, **E** 6-hole rectangular plate **F** Square plate. **(1–3)**: Zygomatic arch fixation methods (lateral view): **1:** Mortice–Tenon structure, **2:** 3-hole plate, **3:** short screw

Then, the fixation methods were reverse-engineered in PTC Creo 4.0. All plates were made of titanium, 2.0 mm in thickness, namely, L-shaped plate (LP) and square plate (SP). Each plate consisted of 4 monocortical screws of 5 mm length, the rectangular plate (RP) consisted of 6 monocortical screws of 5–6 mm length, and the 3-hole plate (3HP) with 1.7 mm thickness and consisted of 3 monocortical screws of 5 mm length was used for zygomatic arch osteotomy line fixation. Additionally, the short screw (SS) was developed at 7 mm in length. Bicortical screws (LS) (2.0 mm) were designed 15 mm in length. Maintaining the freed zygoma in the new position, the six different zygomatic body fixation methods (ZBFm), namely, across the L-shaped osteotomy line’s short-arm: 2LS, LPLS, LP with a short-wing on the maxilla (LPwM), and LP with a short-wing on the zygoma (LPwZ). SP and RP were implanted across the L-shaped osteotomy line’s long-arm. Each ZBFm group combined three different zygomatic arch fixation methods (ZAFm): MT, 3HP, and SS. A total of 18 virtual models were developed, incorporating the virtually operated and fixated zygoma bone and the internal fixation methods (IFM) (Fig. 6).

### Material properties

The zygomatic complex virtual model was constructed of homogenous bone. The bone properties were derived from previous studies, with average values representing normal healthy adult bone [40, 41]. The IFMs had the properties of commercially pure titanium Ti-4AI-6V (Table 4).

**Table 4:** Materials properties used in FEA

Material	Property	Value
Titanium	Elastic modulus^*^	104.8 GPa
	Poisson’s ratio^**^	0.33
	Density	4428.8 kg/m^3^
Bone	Elastic modulus	14.8 GPa
	Poisson’s ratio	0.3
	Density	2000 kg/m^3^

1 GPa (Gigapascal) = 1000 MPa (Pa is the Pascal unit equating to Newton/m^2^)

*Elastic modulus: is the ratio of the stress and strain of an object undergoing elastic deformation

**Poisson’s ratio: represents the ability of a structure to resist deformation in a direction perpendicular to that of the applied load

### Mesh creation and simulation analysis

**Fig. 7:**
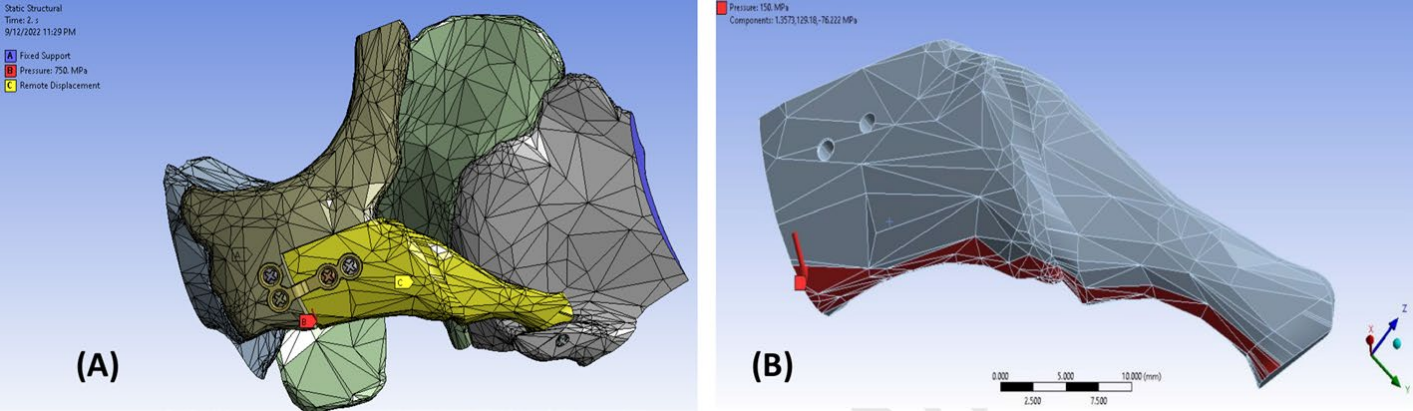
**A** Zygoma bone was the only movable part of the model that could be displaced under the applied pressure, while the surrounding bones were fully restrained, forming the first boundary condition. **B** Force location and direction in the FEA model simulating masseter muscle

ANSYS R19.2 (ANSYS, Inc., Canonsburg, Pennsylvania, U.S.) was used to independently mesh bones, screws, and plates of the assembled model, creating a model consisting of a maximum element size of 3 mm and a minimum of 0.5 mm. The surrounding bone meshed with software default mesh size; only the operated assembled zygomatic bone meshed finer for more accurate results (199,292 elements with 344,199 nodes). The freed zygoma and the surrounding bone contact surfaces were movable and nonpenetrating, while the screws were rigidly fixed to the bone (Fig. 7A). Frictional contact was set between the titanium plate surface and the fixated bone. Zygoma bone was the only movable part under the applied forces, while the surrounding bones were fully restrained, forming the first boundary condition. Then, we depicted the inferior surface of the zygomatic complex (body and arch), simulating the location and direction of the masseteric force representing the second boundary condition (Fig. 7B). The force magnitudes were determined based on the studies of Okiyama et al. [42] and Sarkarat et al. [43] to be 150 N/mm^2^ (average mastication force) and 750 N/mm^2^ (maximal mastication force) along the Z-axis. The remote displacement formed the third boundary condition. In this step, we guided ANSYS to allow the operated zygoma to rotate in all directions and be displaced in the Z and X directions, representing the masseteric inferolateral pulling force. Otherwise, the zygoma bone could not show the displacement and the generated stress.

**Fig. 8:**
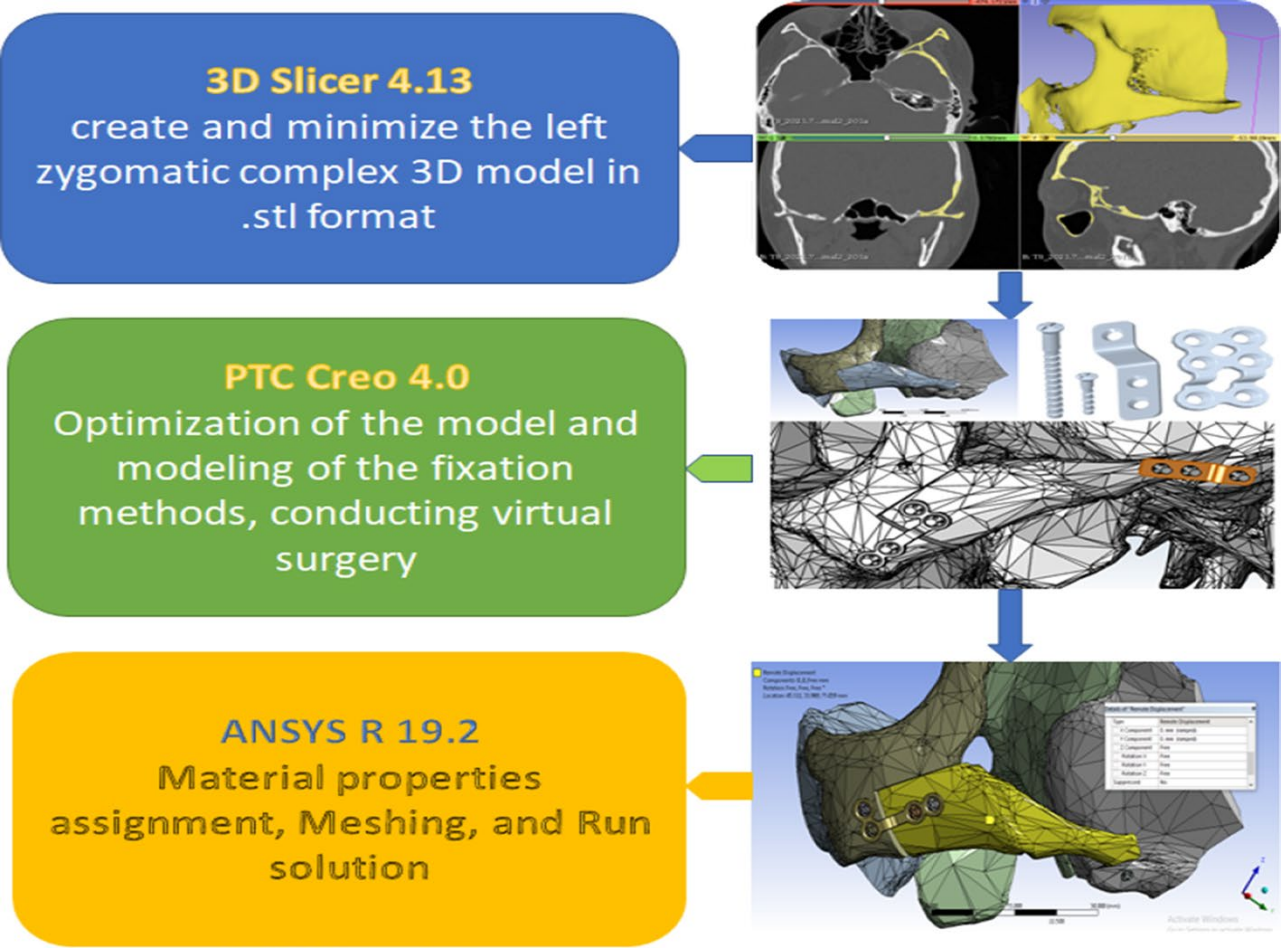
Finite element analysis processing flowchart

Finally, to obtain the required outcomes, we ran the solution after setting up all the models in terms of stress, strain, and displacement registration over the overall model, zygoma bone, and fixation methods, as shown in the flowchart (Fig. 8).

### Statistical analysis

Descriptive statistics were used regarding the average and maximum values of the recorded stress, strain, and displacement. ANOVA was used to reveal the presence of statistically significant differences among the ZBFm and ZAFm groups under the loaded forces. If significant, a post hoc test with Bonferroni correction was used for multiple comparisons. SPSS Statistics version 25 (IBM Corp., Armonk, NY, USA) was used for all analyses. A *P*-value of less than 0.05 was considered statistically significant.

## Data Availability

The data used and/or analysed during the present study are available from the corresponding author on reasonable request.

## Abbreviations

FM: Fixation methods
LORM: L-shaped osteotomy reduction malarplasty
FEA: Finite element analysis
N/mm^2^: Newton/squared millimetre
3D: Three dimensional
2LS: Two bicortical screws
MT: Mortice–tenon
RP: Rectangular plate
SP: Square plate
LPwM: L-shaped plate with short-wing on the maxilla
LPwZ: L-shaped plate with short-wing on the zygoma
LPLS: L-shaped plate with one bicortical screw
RM: Reduction malarplasty
LO: L-shaped osteotomy
3HP: Three-hole plate
SS: Short screw
3D-FEA: Three-dimensional finite element analysis
ZBFm: Zygomatic body fixation methods
ZAFm: Zygomatic arch fixation methods
CT: Computed tomography
DICOM: Digital Imaging and Communications in Medicine
STL: Standard tessellation language
IFM: Internal fixation methods
ANSYS: A general-purpose, finite-element modeling package
ANOVA: Analysis of variance
SPSS: Statistical Package for the Social Sciences
